# Methotrexate Therapy in Juvenile Idiopathic Arthritis: No Clinically Relevant Pulmonary Impairment but Frequent Transient Liver Enzyme Elevations in a Longitudinal Single‐Center Pediatric Cohort of 274 Children Over 30 Years

**DOI:** 10.1002/acr2.90000

**Published:** 2026-03-23

**Authors:** Janosch Weßling, Christoph Leiskau, Frank Dressler, Christian Klemann

**Affiliations:** ^1^ Division of Paediatric Pneumology, Allergology and Neonatology, Department of Paediatrics Hannover Medical School Hannover Germany; ^2^ Department of Pediatrics and Adolescent Medicine, University Medical Center Göttingen Georg August University Göttingen Germany; ^3^ Department of Pediatric Immunology, Rheumatology and Infectiology, Hospital for Children and Adolescents University Hospital Leipzig Leipzig Germany

## Abstract

**Objective:**

Methotrexate (MTX) is a cornerstone in treating juvenile idiopathic arthritis (JIA). The long‐term impact of MTX, particularly on pulmonary and liver function, remains a concern. We longitudinally examined the effects of MTX on pulmonary and hepatic function in the largest reported cohort to date.

**Methods:**

In this retrospective, single‐center study, patients with JIA receiving MTX (10–15 mg/m^2^) between 1993 and 2023 were analyzed. Pulmonary function tests (PFTs), including body plethysmography and diffusing capacity for carbon monoxide (DLCO), were performed annually (±3 months) starting at age 5–6 years by professional lung function technicians according to international guidelines. Hepatic enzymes (glutamate pyruvate transaminase [GPT]/alanine aminotransferase) were assessed quarterly with annual abdominal ultrasounds. Data points were compared using paired *t*‐tests against baseline and prior values.

**Results:**

A total of 274 patients with JIA (189 female patients and 85 male patients) with a mean age at disease onset of 7.8 years were treated with MTX for a mean duration of 42.7 months (maximum 14.3 years), corresponding to 918 treatment‐years. No clinically relevant MTX‐associated lung disease occurred, and spirometric, lung volume, and diffusion parameters remained stable over time. One‐third of the 274 pt hat RANSIENT liver enzyme elevations in liver enzyme levels that resolved after dose adjustment or temporary interruption; only 17 of 63 affected patients required permanent discontinuation due to recurrent transaminase elevations. No structural hepatic pathology was detected. Overall, MTX was discontinued in 59% of patients, most commonly due to sustained remission (53%). Other reasons included GPT elevation, nausea, cytopenia, inefficacy, and noncompliance and accounted for only a small minority of patients.

**Conclusion:**

In children with JIA, long‐term treatment with MTX appears to be safe regarding the pulmonary outcome. Hepatic involvement was frequent but transient, occasionally requiring dose adjustment or discontinuation without long‐term complications.

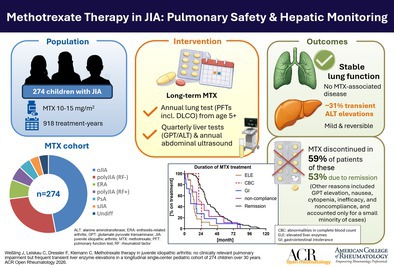

## Graphical abstract

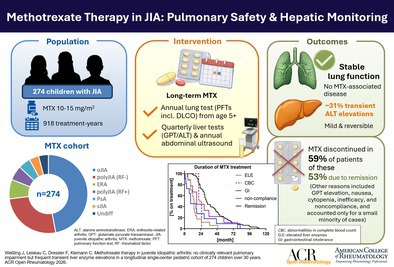

## INTRODUCTION

Juvenile idiopathic arthritis (JIA) is the most common chronic rheumatic disease in childhood, characterized by heterogeneous clinical manifestations and driven by complex genetic and environmental interactions that lead to chronic joint inflammation.

Following a landmark trial in 1992, methotrexate (MTX) became the most prescribed nonbiologic disease‐modifying antirheumatic drug for the treatment of polyarticular JIA.^1^ MTX is also effective in other JIA subtypes such as oligoarticular JIA,^2^, ^3^, ^4^ juvenile psoriatic arthritis, and enthesitis‐related arthritis (ERA) with peripheral joint involvement.^5^, ^6^, ^7^

Despite its widespread use, the long‐term impact of MTX, particularly on liver and pulmonary function, has been a subject of ongoing concern. Hepatotoxicity, manifesting as elevated liver enzymes, is the most common liver‐related side effect, potentially leading to liver fibrosis or cirrhosis with prolonged exposure. The risk of liver damage is influenced by cumulative dose, duration of therapy and preexisting liver conditions.^8^, ^9^ Regular monitoring of liver enzymes is recommended to detect early signs of hepatotoxicity.^10^, ^11^ Management strategies include dose reduction, temporary discontinuation of MTX or switching to alternative therapies, if significant liver enzyme elevations or clinical signs of liver damage occur.^12^, ^13^, ^14^, ^15^ The concern regarding pulmonary toxicity is largely rooted in observations from adult populations, in whom MTX has been associated with MTX‐induced pneumonitis, interstitial lung disease and lung fibrosis.^16^, ^17^, ^18^ In adults, deterioration in pulmonary function tests (PFTs) has been demonstrated after long‐term use, and thus yearly PFT are recommended in adults.^17^, ^18^, ^19^ However, an obstacle in interpreting the finding of deteriorating lung function is the uncertainty of causality: Worsening of lung affection may represent a side effect of MTX treatment or alternatively be caused by the natural course of severe rheumatic disease, and some authors prefer the latter conclusion, inferring it to be due to increased systemic inflammation.^20^, ^21^

In contrast, data on the pulmonary side effects of MTX in pediatric populations, especially in those with JIA, remain limited and inconclusive. Given the critical developmental stages during childhood and adolescence, understanding the potential pulmonary implications of long‐term MTX therapy in this demographic is of paramount importance.^22^, ^23^, ^24^

An overview of relevant clinical studies on MTX‐related pulmonary and hepatic side effects in children with JIA is provided in Supplement Tables 1 and 2. These tables offer a structured summary of previous findings, highlighting disease‐ and treatment‐associated alterations in lung and liver function. Supplement Table 1 underscores the nuanced effects of MTX on pulmonary function in patients with JIA, emphasizing the critical importance of further investigation of the effect of long‐term MTX treatment on lung function in children with JIA. Therefore, we retrospectively performed a longitudinal analysis of the last 30 years in a single‐center pediatric cohort of 274 patients receiving MTX, with a special focus on hepatic and lung involvement.

## PATIENTS AND METHODS

### Patients

The retrospective, explorative study in accordance with the Declaration of Helsinki was approved by the local ethics committee (Hannover Medical School, Germany; permit number 10050_BO_K_2021). Relevant patients’ data were collected by searching and analyzing printed patients’ files from 2007 to 2023 and by using the hospital's SAP software.

Inclusion criteria were a definite diagnosis of JIA and MTX treatment for at least one year. A total of 274 children receiving MTX at a dose of 10 to 15 mg/m^2^ of body surface area were identified between 1993 and 2023. The study period covered a total of 30 years, representing the overall observation window of our center. Individual follow‐up durations varied between 1 and 16 years with a mean observation period of approximately five years per patient. MTX was initiated at the full target dose of 10 to 15 mg/m^2^ without gradual dose escalation, with liver function tests performed after four weeks and subsequently at three‐month intervals. The first 68 patients from the years 1993 to 2007 have been previously reported by our group (45 female patients and 23 male patients).^25^ Furthermore, 206 children (146 female patients and 60 male patients) were treated with MTX between 2007 and 2023.

Patients with asthma were not excluded from the analysis, as this reflects real‐world clinical practice. Bronchopulmonary chronic or structural pulmonary disease other than asthma (eg, cystic fibrosis, bronchiopulmonary dysplasia, interstitial lung disease unrelated to JIA or congenital malformations) would have been excluded, but no such patients were present in our cohort.

MTX was initiated in oligoarticular JIA when one or more of the following were present: extended oligoarthritis, persistent oligoarthritis with poor response to intra‐articular glucocorticoids, recurrent synovitis in previously injected joints, or extra‐articular manifestations (eg, chronic anterior uveitis requiring systemic immunosuppression).

### PFTs

Pulmonary function tests (PFTs), including body plethysmography and diffusing capacity for carbon monoxide (DLCO), were performed annually (±3 months) starting at age 5–6 years by professional lung function technicians according to international guidelines.^26^, ^27^ Until 1997, body plethysmography was performed with “Body‐Ganzkörperplethismograph” by “Fenyves+Gut”; after 1998, body plethysmography was performed with “Body‐Body‐Scope N” by “Ganshorn Medizin Electronic GmbH” and “Master Screen Body by Jaeger/Care Fusion”; and since 2014, body plethysmography has been performed with “PowerCube Body+” by “Ganshorn Medizin Electronic GmbH.” Until 2016, DLco was performed with “Master Screen Diffusion” by “Jaeger/Care Fusion,” and since 2016, DLco has been performed with “PowerCube‐Diffusion+” by “Ganshorn Medizin Electronic GmbH.”

Extracted parameters of interest were forced expiratory volume in the first second (FEV1), forced vital capacity (FVC), maximal mid‐expiratory flow (MMEF), total lung capacity (TLC), residual volume (RV), and DLco. Values are provided as a percentage of reference values that account for age, sex, and height using Global Lung Function Initiative (GLI) reference standards.

### Hepatic side effects

Liver enzyme data (glutamate pyruvate transaminase [GPT]/alanine aminotransferase [ALT] elevation) were collected by analyzing the SAP software and by searching the archive data of Hannover Medical School (ALIDA software). Concomitantly with annual PFT, abdominal ultrasound scans were systematically performed to evaluate potential hepatic damage attributable to MTX therapy. With this approach we were able to monitor liver health, including the identification of steatosis, fibrosis, or any morphologic changes indicative of liver disease. The analysis of the hepatic side effects was limited to the cohort from 2007 to 2023.

### Statistics

The statistical evaluation was conducted in collaboration with the Institute for Biometrics at Hannover Medical School to ensure methodologic rigor. Given the longitudinal design, lung function parameters were analyzed using paired *t*‐tests to compare baseline values with subsequent measurements. Percentages based on normative values adjusted for age, weight, and height were used to account for developmental differences in pulmonary function. Exploratory analyses were performed for different JIA categories, applying analogous statistical methods. As this was an exploratory study, no multiplicity correction was applied. *P* < 0.05 was considered significant. Statistical analyses were conducted using GraphPad Prism, version 9.0. A large language model (ChatGPT 5.0) was used for polishing the English.

## RESULTS

This study subsumes 274 patients (189 female patients and 85 male patients) who were treated weekly with MTX in a standard dose of 10 to 15 mg/m^2^. The mean dose at initiation was 13.6 ± 1.9 mg/m^2^ with a median of 14 mg/m^2^. Minor dose adjustments occurred during follow‐up, apart from temporary reductions in patients with elevated liver enzyme levels. The mean age at disease onset requiring MTX treatment was 7.8 years, with a median of 7.0 years (range 0.8–17.4 years). The mean duration of MTX treatment was 42.7 months (median 32.5 months). The longest analyzed treatment duration was 14.2 years. In total, we oversaw 918 years of MTX treatment in patients with JIA (Supplement Table 3).

**Figure 1:**
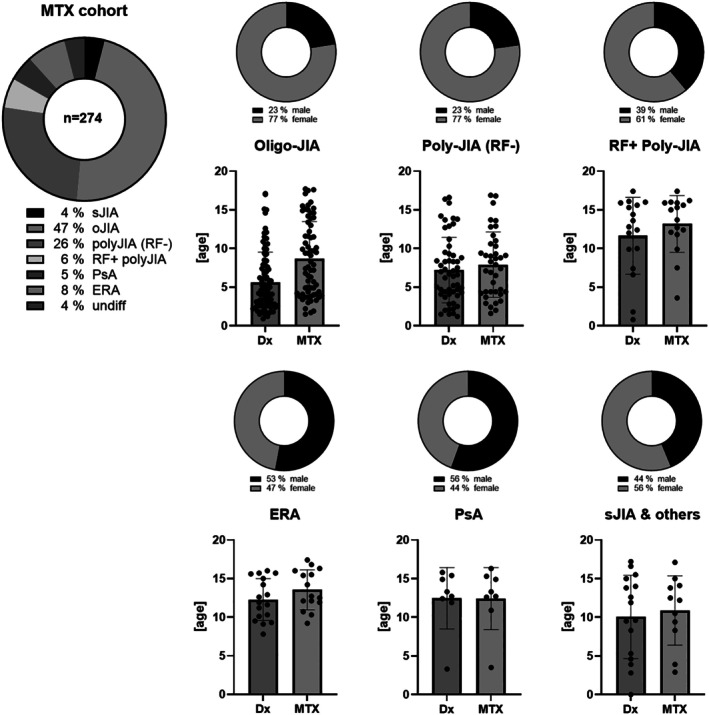
Demographic and clinical characteristics of pediatric JIA cohort treated with MTX encompassing 274 children between 1993 and 2023. This figure presents a comprehensive overview of the distribution across JIA subgroups, along with the sex and age at onset, among children who received MTX treatment at our facility within the specified timeframe. The donut chart on the left shows the distribution of JIA subtypes in 274 patients. The other donut charts display sex distribution per subtype. Scatter plots with mean ± SD compare age at Dx versus age at MTX initiation for each JIA subtype. Dx, diagnosis; ERA, enthesitis‐related arthritis; JIA, juvenile idiopathic arthritis; MTX, methotrexate; oJIA, oligoarthritis; polyJIA (RF−), polyarticular JIA with negative rheumatoid factor; RF+ polyJIA, polyarticular JIA with positive rheumatoid factor; PsA, psoriatic arthritis; sJIA, systemic JIA (Still disease); undiff, undifferentiated JIA.

The predominant JIA category within our cohort was oligoarthritis, encompassing 130 patients (47.4%) (Figure 1). The next most prevalent category was polyarticular JIA with negative rheumatoid factors (RFs), accounting for 71 patients (25.9%), followed by ERA in 21 patients (7.7%) and polyarticular JIA with positive RFs in 16 patients (5.8%). Psoriatic arthritis and systemic JIA (sJIA) were less common, comprising 14 (5.1%) and 11 (4%) patients, respectively. The low proportion of patients with sJIA reflects real‐world MTX use, as biologic therapies are preferred first‐line in this JIA category. An additional 11 patients (4%) were classified with an indeterminate or unspecified category of JIA (Figure 1). Therefore, the distribution of JIA categories in our cohort deviates from the expected normal distribution of JIA subsets, primarily due to the specific inclusion criterion of MTX treatment.

### Pulmonary function parameters

**Figure 2:**
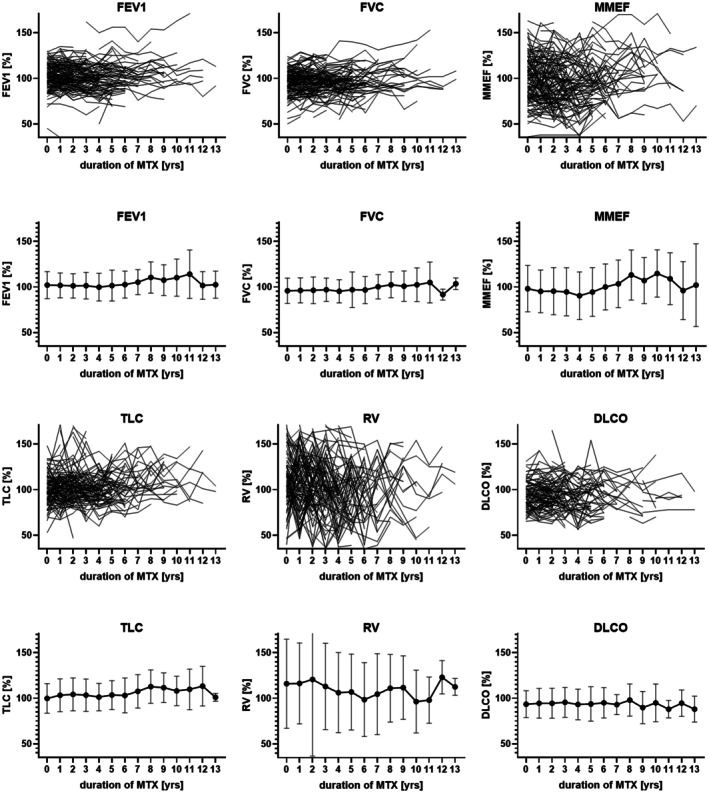
Upper row: each line represents a single patient's individual longitudinal development of lung function values in the percentage of the age‐specific normal values (y‐axis) throughout MTX treatment in years (x‐axis). Lower row: mean values (+ SD) of age‐specific lung function (y‐axis) in the cohort of 274 patients related to treatment duration (x‐axis) in years. Paired *t*‐test comparing each time point with the lung function before starting MTX showed no significant changes. DLco, diffusing capacity for carbon monoxide; FEV1, forced expiratory volume in the first second; FVC, forced vital capacity; JIA, juvenile idiopathic arthritis; MMEF, maximal mid‐expiratory flow; MTX, methotrexate; RV, residual volume; TLC, total lung capacity.

The analysis of PFT parameters among patients indicated that the majority of measured values fell within the range of 88.0% to 123.0%, with the normative lower threshold for these parameters established at <80%. The PFT parameters evaluated included FEV1, FVC, MMEF, TLC, RV, and DLco. Longitudinal monitoring of these parameters did not demonstrate any significant variations over time (Figure 2).

**Figure 3:**
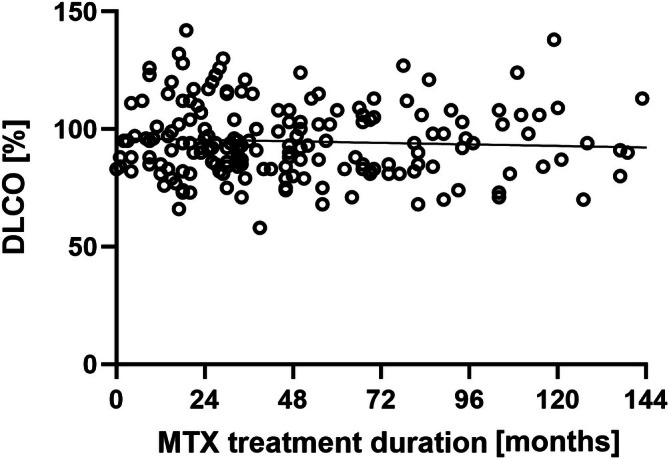
Scatter plot illustrating the relationship between the duration of MTX treatment (in months) and the DLco, expressed as a percentage of the predicted age‐adjusted normal value. Each circle represents an individual patient. The nearly flat regression line indicates no apparent trend, consistent with the statistical analysis showing a very weak, nonsignificant correlation (r = −0.06129, *P* = 0.4111). These data suggest that MTX treatment duration does not meaningfully impact DLco in this cohort. DLco, diffusing capacity for carbon monoxide; MTX, methotrexate.

At baseline (time point 0), mean PFT values for FEV1, FVC, and TLC approximated 100% of the age‐, sex‐, and height‐specific normative values, aligning with expectations for a healthy pediatric population (Supplement Tables 3 and 4). Conversely, RV exhibited a slight elevation above expected levels, whereas DLco was marginally reduced at approximately 93% compared to the reference population (Supplement Tables 3 and 4). Despite these deviations, both RV and DLco remained within the physiologic range, indicating no significant pulmonary compromise at the onset of MTX treatment (Figures 2 and 3). Throughout the observation period extending up to 14 years, no significant longitudinal alterations were observed in PFT parameters (FEV1, FVC, MMEF, TLC, RV, and DLco) among the patients receiving MTX therapy (Figures 2 and 3). The cohort size diminished progressively over time, attributable to the discontinuation of MTX in the patients or reaching adulthood with subsequent loss of follow‐up. The reduction in patient numbers over the years was primarily due to the achievement of clinical remission with only a minor proportion discontinuing due to MTX‐induced nausea or elevated liver enzyme levels. No patient exhibited clinical evidence of pulmonary disease at their last assessment.

To further explore whether prolonged MTX therapy may be associated with impaired pulmonary function, the latest DLco value of each patient was analyzed against the treatment length. Pearson correlation analysis revealed no significant linear relationship between the duration of MTX treatment and the DLco, expressed as a percentage of the predicted value. The correlation coefficient (r = −0.06) indicated a very weak negative association, with a nonsignificant *P* value (*P* = 0.41). These findings suggest that MTX treatment duration does not impact DLco in this cohort (Figure 3).

To investigate whether lung function trajectories during MTX treatment vary across different categories of JIA, a subgroup analysis was conducted based on the International League of Associations for Rheumatology (ILAR) classification. This demonstrated stable trends across all lung function parameters within each JIA category, with no MTX‐associated longitudinal decline observed. These findings suggest that MTX treatment does not lead to clinically meaningful changes in pulmonary function over time in any of the analyzed JIA categories (Supplement Figure 1).

Although no relevant changes in pulmonary function were observed over the longitudinal course of MTX therapy at the group level over time, a small subset of 12 patients demonstrated notable deviations from predicted values in PFTs. In one patient, PFT parameters were persistently below the expected range due to limited test compliance related to the patient's condition of mutism, which was unrelated to her JIA diagnosis. Another patient treated with MTX for four years exhibited a constant borderline reduction in DLco, whereas TLC remained within the normal range. Clinical features, including a chronic dry cough and recurrent respiratory infections, raised suspicion of interstitial lung disease; however, further diagnostics did not prove any lung pathology. In a similar patient with suspected interstitial lung disease, the frequency of PFT assessments was increased. In this patient, TLC also remained within normal limits, and no diagnosis of interstitial lung disease could be made. MTX therapy was ultimately tapered and discontinued after disease remission, and lung function parameters remained stable. Poor test compliance or subclinical stable reductions without corresponding respiratory symptoms were attributed to reduced PFT values.

### Hepatic side effects

In our institution, we initiated MTX therapy at a standard dose of 10 to 15 mg/m^2^ body surface area. Liver function tests (LFTs), including ALT/GPT, were first performed four weeks after treatment initiation, followed by quarterly monitoring thereafter.

Transient elevations in liver enzymes were observed as the only hepatic side effect. ALT levels exceeding twice the upper limit of normal occurred in 63 patients (30.6%), with 57 of these patients surpassing a three‐fold ALT elevation. Importantly, no patient of irreversible liver injury or clinically significant hepatic fibrosis was identified. Abdominal ultrasound examinations were performed annually and revealed no signs of chronic liver pathology.

**Figure 4:**
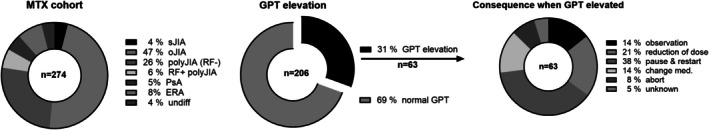
Multifaceted analysis of JIA subgroups and MTX‐associated hepatotoxicity, 1993 to 2023. (Left panel) Categorization and distribution of JIA subtypes treated with MTX at our institution between 1993 and 2023, including sJIA, oJIA, polyJIA (RF−), RF+ polyJIA, PsA, and ERA. (Middle panel) Incidence of GPT level elevation observed in this cohort (2007–2023), indicative of potential liver enzyme abnormalities. (Right panel) Overview of the clinical implications and management strategies adopted in response to GPT level elevation, highlighting the hepatotoxic risk associated with MTX therapy in this population. ERA, enthesitis‐related arthritis; GPT, glutamate pyruvate transaminase; JIA, juvenile idiopathic arthritis; MTX, methotrexate; oJIA, oligoarthritis; polyJIA (RF−), polyarticular JIA with negative rheumatoid factor; PsA, psoriatic arthritis; RF+ polyJIA, polyarticular JIA with positive rheumatoid factor; sJIA, systemic JIA (Still disease); undiff, undifferentiated JIA.

Among the 63 patients with elevated ALT levels, management was individualized according to the severity and clinical context as presented in Figure 4. In 9 of 63 patients (14%), a watchful waiting approach without dose modification led to spontaneous normalization. MTX dose reduction was implemented in 13 children (21%), whereas 24 children (38%) underwent a temporary treatment pause of two to four weeks. A therapeutic switch, most commonly to tumor necrosis factor inhibitors, was necessary in nine patients (14%) among those with elevated ALT levels (n = 63). MTX was permanently discontinued in eight patients (12%) without the need for switching therapy due to sustained remission. Taken together, these findings highlight that MTX‐associated liver enzyme level elevations were generally mild, reversible, and manageable without long‐term hepatic complications.

### Other reasons for MTX discontinuation

Among the 274 children receiving MTX, 161 patients (58.8%) ultimately discontinued therapy during the observation period. Fortunately, the most common reason for cessation was achievement of stable clinical remission, accounting for about half of all discontinuations (82/161; 52.6%). Intolerance‐related factors constituted a smaller but relevant proportion (Supplement Figure 2): recurrent transaminase level elevations as mentioned earlier in 17 patients (10.9%), nausea leading to discontinuation in 10 patients (6.4%), and cytopenias (CBC abnormalities) in 2 patients (1.3%). MTX discontinuation due to treatment inefficacy was documented in five patients (3.2%). Noncompliance represented another infrequent cause (n = 9; 5.8%). In 30 patients (19.2%), documentation was insufficient to determine the exact reason for stopping MTX. Overall, these findings underscore that the majority of MTX discontinuations occurred due to remission rather than drug‐related toxicity or inefficacy, reflecting the generally favorable long‐term tolerability of MTX in this pediatric JIA cohort.

## DISCUSSION

Despite the widespread use of MTX in children with JIA, data on long‐term pulmonary safety remain limited. Our longitudinal study of 274 patients over 918 treatment‐years—the hitherto largest longitudinally analyzed pediatric MTX cohort—demonstrates overall stable lung function and no evidence of clinically relevant pulmonary or permanent hepatic toxicity during treatment.

The cohort was predominantly composed of patients with oligoarthritis (47%) and RF‐negative polyarthritis (26%), reflecting a selection bias depending on MTX treatment. JIA categories like sJIA and enthesitis‐related arthritis(ERA), which are less responsive to MTX, were underrepresented (Supplement Table 3, Figure 1). Due to the low number of patients with sJIA, our findings regarding pulmonary safety cannot be extrapolated to sJIA‐associated lung disease (sJIA‐LD), which is mainly driven by disease‐specific inflammation rather than MTX exposure.

PFTs, including FEV1, FVC, TLC, RV, MMEF, and DLco, remained stable throughout the observation period. Patients withdrawn from longitudinal lung function analysis had either achieved clinical remission or transitioned to adult care; none exhibited clinical evidence of pulmonary disease at their last assessment. As shown in the longitudinal trends across the cohort and supported by individual patient trajectories, MTX treatment did not cause any clinically meaningful decline in pulmonary function (Figure 2, Supplement Table 4). Additionally, we found no correlation between MTX treatment duration and DLco, further supporting the absence of cumulative pulmonary toxicity (Figure 3). This was consistent across all JIA categories, as illustrated in the category‐specific analyses (Supplement Figure 1), reinforcing that neither disease category nor MTX exposure were associated with progressive lung function impairment aligning with previous studies.^22^, ^28^, ^29^, ^30^

In contrast, cross‐sectional studies such as those by Attanasi et al^24^ and Pelucchi et al^23^ have reported reduced DLco and MMEF in MTX‐treated patients. Lucantoni et al found significant impairment of DLco in patients with JIA treated with MTX compared to controls.^31^ However, these findings are limited by small sample sizes and confounded by disease activity and lack of longitudinal data. Our study design mitigates these issues, allowing for temporal assessment and more accurate attribution of changes in pulmonary function. Rare case reports, including those by Liu et al^32^ and Cron et al,^33^ describe MTX‐induced pneumonitis in children. These underscore the need for clinical vigilance but do not support routine pulmonary screening in asymptomatic patients. Exceptions may apply to selected high‐risk groups. One such group includes children with sJIA, who—though infrequently treated with MTX—are at risk for developing sJIA‐LD, a distinct entity characterized by radiologic and histopathologic changes often linked to macrophage activation syndrome and elevated interleukin‐18 levels, previously described by Schulert et al.^34^ This was also investigated by Petrongari et al^35^ emphasizing the need for enhanced understanding and the development of tailored therapeutic approaches in children with sJIA. Although our data support the notion that MTX is not causative, we recommend routine PFTs for children with Still disease (regardless of MTX exposure) given their heightened baseline risk of interstitial lung disease. Furthermore, adolescents with RF‐positive polyarticular JIA are at increased risk of progression to adult rheumatoid arthritis.^36^ For these patients, we recommend periodic lung function testing, particularly in the presence of high cumulative MTX exposure, as a precautionary measure during adolescence and transition to adult care. Taken together, our findings confirm the pulmonary safety of MTX in children with JIA. Routine PFT monitoring may not be necessary for all patients but should be considered in high‐risk subgroups, such as those with Still disease or RF‐positive polyarthritis regardless of MTX treatment.

Elevations in liver enzyme levels are among the most common adverse effects associated with MTX therapy. In our cohort, 30.6% of patients experienced transient alanine transaminase (ALT/GPT) elevations above twice the upper limit of normal, with 90% exceeding a three‐fold increase. Despite this relatively high frequency, no cases of irreversible liver damage or structural pathology were observed by the yearly sonography checkups and enzyme levels normalized in all affected patients with appropriate dose adjustments or (temporary) discontinuation. Our findings contrast with lower rates reported in adult RA populations, such as the 6.4% observed by Dubey et al,^15^ which may reflect age‐related differences in metabolism, disease burden or surveillance intensity. In line with pediatric data from Lahdenne et al,^12^ who found only mild histologic changes under low‐dose MTX, our results support the safety of MTX at standard pediatric dosing (10–15 mg/m^2^). Most hepatic events in our cohort were managed conservatively and only 10.9% required permanent MTX discontinuation. These discontinuation rates highlight that MTX intolerance and inefficacy were uncommon, supporting the long‐term tolerability of MTX in real‐world pediatric JIA cohorts. This is notably lower than discontinuation rates reported by Barral Mena et al^13^ in pediatric JIA. In addition, international registry data such as the Pharmachild dataset, which includes 6,963 patients with JIA treated with MTX, reported 595 gastrointestinal and 233 hepatobiliary adverse events.^37^ Compared with our observed frequency of transient hepatic enzyme elevations (30.6%), the 3.3% hepatobiliary adverse events rate reported in Pharmachild likely reflects divergent definitions (biochemical abnormalities vs coded adverse events) underscoring the complementary nature of our real‐world, long‐term dataset.

The low switch rate to alternative DMARDs reflects our close monitoring and conservative management strategy, in which transient liver enzyme elevations were handled by dose reduction or brief interruption rather than immediate drug discontinuation. Compared with registry data such as Raab et al,^3^ our cohort showed a similar overall discontinuation rate. Thus, our findings support the continued use of MTX with appropriate liver function monitoring in line with current pediatric practice. Importantly, our clinical protocol—starting MTX at the full recommended dose without stepwise escalation and conducting the first liver enzyme check after 4 weeks, followed by testing every 12 weeks—proved to be both feasible and safe. This approach streamlined treatment initiation without compromising hepatic safety. Of note, MTX‐related hepatotoxicity was not confined to a specific JIA category, though patients with juvenile psoriatic arthritis showed a slightly higher rate of enzyme level elevations, consistent with findings by Tilling et al in adult psoriatic arthritis.^38^ We did not assess pharmacogenetic risk factors such as the SLCO1B1 variant described by Roszkiewicz et al,^39^ which may help refine risk stratification in future studies.

It should also be noted that transient liver enzyme elevations may in some cases be unrelated to MTX exposure but occur secondary to intercurrent viral or bacterial infections, which are common in pediatric patients undergoing routine laboratory monitoring. This should be considered a relevant confounder for liver enzyme fluctuations in our cohort.

In summary, our data indicate that hepatic side effects of MTX in children with JIA are common but largely transient and manageable. Regular monitoring remains essential, particularly in categories with known susceptibility or when transitioning into adolescence, where cumulative exposure increases.

Our study faced several limitations, including the retrospective, single‐center design; variable follow‐up durations; declining case numbers over time; incomplete dosing documentation in early years; and limited representation of enthesitis‐associated JIA and sJIA due to MTX treatment being an inclusion criterion. Missing PFTs in younger children may also introduce bias. Nevertheless, consistent monitoring protocols and a large number of MTX treatment years strengthen the validity of the findings.

This 30‐year longitudinal study represents the largest pediatric cohort to date assessing MTX safety in JIA. Our findings confirm that long‐term MTX therapy is well‐tolerated with respect to both pulmonary and hepatic outcomes. Lung function remained stable across all JIA categories, and no clinically significant pulmonary toxicity occurred. Hepatic enzyme elevations were frequent but transient and rarely necessitated permanent discontinuation. Our approach of initiating MTX at the full recommended dose without gradual escalation, combined with liver enzyme monitoring starting at 4 weeks and every 12 weeks thereafter, proved to be both safe and feasible in routine pediatric care.

Based on our data, routine PFT is not required for all MTX‐treated children. However, we recommend baseline and follow‐up lung function testing in selected high‐risk groups, particularly adolescents with RF‐positive polyarthritis—due to the potential for transition to adult rheumatoid arthritis—and all patients with sJIA (Still disease), given their elevated baseline risk for interstitial lung disease. With these targeted measures, MTX remains a safe and effective cornerstone therapy in the long‐term management of JIA.

## AUTHOR CONTRIBUTIONS

All authors contributed to at least one of the following manuscript preparation roles: conceptualization AND/OR methodology, software, investigation, formal analysis, data curation, visualization, and validation AND drafting or reviewing/editing the final draft. As corresponding author, Dr Klemann confirms that all authors have provided the final approval of the version to be published and takes responsibility for the affirmations regarding article submission (eg, not under consideration by another journal), the integrity of the data presented, and the statements regarding compliance with institutional review board/Declaration of Helsinki requirements.

## Supporting information


**Disclosure Form**:

**Data S1.** Supporting Information.
