# Correction to “Methotrexate Therapy in Juvenile Idiopathic Arthritis: No Clinically Relevant Pulmonary Impairment but Frequent Transient Liver Enzyme Elevations in a Longitudinal Single‐Center Pediatric Cohort of 274 Children Over 30 Years”

**DOI:** 10.1002/acr2.90058

**Published:** 2026-04-21

Weßling, J. , Leiskau, C. , Dressler, F. and Klemann, C. (2026), Methotrexate Therapy in Juvenile Idiopathic Arthritis: No Clinically Relevant Pulmonary Impairment but Frequent Transient Liver Enzyme Elevations in a Longitudinal Single‐Center Pediatric Cohort of 274 Children Over 30 Years. ACR Open Rheumatology, 8: e90000. 10.1002/acr2.90000 41873148 PMC13093451

In the results section of the abstract, it says “One‐third of the 274 pt hat RANSIENT liver enzyme elevations in liver enzyme levels that resolved after dose adjustment or temporary interruption; only 17 of 63 affected patients required permanent discontinuation due to recurrent transaminase elevations.”

This should have read: “One‐third of the cohort developed transient elevations in liver enzyme levels that resolved after dose adjustment or temporary interruption; only 17 of 63 affected patients required permanent discontinuation due to recurrent transaminase elevations.”

We apologize for this error.

