# Supplementary material for: Methotrexate Therapy in Juvenile Idiopathic Arthritis: No Clinically Relevant Pulmonary Impairment but Frequent Transient Liver Enzyme Elevations in a Longitudinal Single‐Center Pediatric Cohort of 274 Children Over 30 Years
Source: ACR Open Rheumatol. 2026 Mar 23;8(3):e90000. doi: 10.1002/acr2.90000 (PMC13093451; doi:10.1002/acr2.90000)
Supplement: Supplementary file 2 — Data S1. Supporting Information. [file ACR2-8-e90000-s001.docx]

**Supplementary Material**

**Supplement Table 1.**

| **Study** | **Study design** | **Patients** | **Key Finding** | **Conclusion** |
| --- | --- | --- | --- | --- |
| “Pulmonary function in juvenile rheumatoid arthritis” – Wagener et al., 1981 (40) | Cross-sectional study | 16 JIA-patients + 37 controls | - 6 with normal PFT results - 2/3 of polyartiular and ½ of pauciarticular with abnormal lung function - 7/15 with low DLCO / 5 remained low - 1 patient with abnormal chest X-ray (pulmonary nodule) - 2 smokers | The study examined pulmonary function in JIA (JRA) patients not linked to MTX.  Found abnormalities including decreased airflow, lung volume, gas diffusion and ventilation-perfusion mismatch during exercise.  Abnormal diffusion capacities indicating possible alveolar capillary interface abnormalities (significant proportion of patients)  -- > Previously unrecognized pulmonary involvement in JIA/JRA |
| “Morbidity associated with long-term methotrexate therapy in juvenile rheumatoid arthritis” - Graham et al., 1992 (28) | Longitudinal study | 62 JIA-children, polyarticular | - No reduction in diffusing capacity or development of restrictive indexes. - No MTX induced pneumonitis was reported - Clinical outcome: No patient had to stop MTX therapy permanently | Methotrexate appears to be well tolerated by the pediatric population, with infrequent morbidity. |
| “Lung function and diffusing capacity for carbon monoxide in patients with juvenile chronic arthritis: effect of disease activity and low dose methotrexate therapy” – Pelucchi et al., 1994 (23) | Cross-sectional study | 61 JIA-patients | - MMEF significantly reduced in patients with active disease; - Mean DLCO value reduced (67 + 80 %) - no evidence of any effect of MTX treatment on the pulmonary parameters | JCA is characterized by an impairment of lung function, mainly involving the small airways, and by interstitial damage. These changes are related to the clinical subtypes of the disease and to disease activity. |
| “Methotrexate-induced hypersensitivity pneumonitis in a child with juvenile rheumatoid arthritis” – Cron et al., 1998 (34) | Case report | 1 child, RF-, ANA+ , polyarticular with MIP | - 11-year old girl with JIA - Respiratory symptoms that resolved when MTX-therapy was stopped and corticosteroids given | MTX-induced hypersensitivity pneumonitis is rare but serious complication in children with JIA. Discontinuation and corticosteroid treatment is crucial. Importance of physician awareness. |
| “Lung function abnormalities and respiratory muscle weakness in children with juvenile chronic arthritis” – Knook et al., 1999 (41) | Cross-sectional study | 31 children with polyarticular and systemic JCA + 32 controls  (23 polyarticular, 9 systemic) | - Peak expiratory flow (PEF) and forced vital capacity (FVC) significantly reduced in JCA patients, when compared to reference values - Maximum inspiratory (PI,max) and expiratory (PE,max) pressures were significantly reduced in patients compared to paired control subjects - Respiratory muscle function compromised in JIA-patients | Disease activity and disability: Long-term methotrexate (MTX) treatment correlated significantly with disease activity and disability.  Significant correlations found between lung function parameters, respiratory muscle function, and disability scores, suggesting a link between respiratory impairment and disease severity. |
| “Pulmonary function in children with juvenile idiopathic arthritis and effects of methotrexate therapy” – Schmeling et al., 2002 (22) | Cross-sectional study | 89 children, 40 treated with MTX | - Moderate airway obstruction in two children with known bronchial asthma - No obstructive or restrictive alteration of ventilation in any other patient - Two JIA patients with reduced CO diffusion capacity of 64 and 67 % (one treated with MTX) | - Long-term low-dose methotrexate treatment (up to 3.5 g over several years) appears safe for lung function impairment. - Rare occurrence of lung function impairment in children with juvenile idiopathic arthritis compared to adult rheumatoid arthritis patients. - no significant lung function concerns, even with extended therapy duration. |
| “Lung function in children and adolescents with juvenile idiopathic arthritis during long-term treatment with methotrexate: a retrospective study” – Leiskau et al., 2012 (25) | Longitudinal study | 68 JIA-patients | - Subgroup of 37 patients had PFT before MTX; significant decrease in MMEF after 3 years - DLCO reduced after third year, MMEF decreased between years 3 and 4, FEV1 slightly rose between years 4 and 5 - no significant changes in other parameters. - No correlation between PFT results, cumulative MTX dose, or JIA subtype. - No clinically relevant lung disease in any patient. | MTX therapy showed relative safety in JIA patients; no correlation found between PFT results and MTX dose or JIA subtype. |
| “PReS-FINAL-2077:Lung function evaluation in a juvenile idiopathic arthritis cohort” – Machado Vaz et al. , 2013 (29) | Cross-sectional study | 41 patients, age 6-20 | - No previous known lung diseases - Most PFTs normal, DLCO 93,1% ± 14,2 (no patient had changes in DLCO) - 3 patients with lung function changes (2 restrictive, 1 obstructive spirometry patterns) | Low prevalence of lung function changes. Impairment of lung function rarer than in RA patients. |
| “Life-threatening pneumonitis complicating low-dose methotrexate treatment for juvenile idiopathic arthritis in a child” – Liu et al., 2014 (33) | Case report | 1 JIA-patient, 13 year-old girl | - JIA patient (rheumatoid factor-negative, antinuclear antibody-negative, extended oligoarticular JIA) developed fever, cough, dyspnea after a year of MTX therapy -> severe respiratory distress - CT revealed ground-glass opacities, lung biopsy confirmed interstitial pneumonitis with diffuse alveolar damage | necessity for pediatricians to be continuously vigilant for early nonspecific symptoms of methotrexate pneumonitis  Evaluation+ early recognition |
| “AB1001 Diffusing Capacity for Carbon Monoxide (DLCO) in a Group of Children with Juvenile Idiopathic Arthritis: A Case Control Study (Preliminary Data)” – Lucantoni et al., 2015 (31) | Case-control study | 26 JIA patients, control group of 15 healthy children | - DLCO values significantly reduced in JIA patients in comparison to controls - JIA patients with MTX treatment had worse DLCO values than controls - DLCO significantly lower in patients with active disease | Significant impairment of DLCO in JIA patients compared to controls. MTX treatment and active disease are worsening the results. |
| “Methotrexate in juvenile idiopathic arthritis: advice and recommendations from the MARAJIA expert consensus meeting” – Ferrara et al., 2018 (1) | Literature review | 843 references -> 209 relevant references  (33 clinical trials, 51 reviews, 1 Cochrane metanalysis, 124 other types) | - MARAJIA (Methotrexate Advice and Recommendations on Juvenile Idiopathic Arthritis Expert Meeting): e.g. (…) - MTX as first-line treatment for oligoJIA and polyarticular JIA - also recommended in systemic arthritis with predominant joint inflammation - MTX has shown efficacy in 65-90% of patients - MTX dosages of 10-15 mg/m2/week are recommended | Balanced and evidence-based recommendations 🡪 easier reliable clinical management |
| “Lung function in children with juvenile idiopathic arthritis: A cross-sectional analysis” – Attanasi et al., 2019 (24) | Cross-sectional study | 49 JIA-patients, 70 controls | - DLCO significantly different between JIA children and controls - No differences in FEV1, FVC, FEF25-75, peak expiratory flow, total lung capacity, and residual volume - Significant difference in DLCO among JIA patients treated with MTX compared to other drugs and controls - Negative correlation of DLCO with MTX cumulative dose and treatment duration | MTX treatment shows a dose-dependent effect on lung function.  abnormalities in lung function, even in asymptomatic patients, might be a complication of JIA  🡪assessment of respiratory system (especially DLCO) |
| “Lung clearance index and diffusion capacity for CO to detect early functional pulmonary impairment in children with rheumatic diseases” – Hildebrandt et al., 2021 (32) | Cross-sectional study | 19 patients with rheumatic disease, 12 with JIA | - 2 patients within physiological limits, 8 with elevated LCI and physiological DLCO, 9 with elevated LCI and DLCO indicating pulmonary impairment | LCI is more sensitive than DLCO for detection of early pulmonary cheanges.  Changes may already be present in children with rheumatic disease even without clinical signs of pulmonary disease.   - Suggestion: Integrate LCI in routine follow-up for children with rheumatic diseases |
| “Lung Involvement in Systemic Juvenile Idiopathic Arthritis: A Narrative Review” – Petrongari et al., 2022 (36) | Review |  | - sJIA exhibits unique clinical manifestations, complications, and treatment options - Severe and potentially life-threatening chronic lung diseases (LD) such as pulmonary alveolar proteinosis (PAP), interstitial lung disease (ILD), and pulmonary hypertension (PH) are increasingly observed in children with sJIA. | clinical features, complications, and emerging challenges in the management of systemic juvenile idiopathic arthritis (sJIA), emphasizing the need for enhanced understanding and tailored therapeutic approaches |
| “Methotrexate does not affect lung function in children with juvenile idiopathic arthritis” – Perrin et al., 2023 (30) | Cross-sectional study | 14 JIA-patients receiving MTX treatment for at least 6 months | - No correlation observed between lung volumes, expiratory flow or LCI2,5 and MTX exposure , no correlation between LCI2,5 and DLCOc - DLCO measurements available for 13 patients, normal measurements, no significant correllation found | No relationship between MTX treatment duration or cumulative dose and PFT results.  No evidence of MTX toxicity in children with JIA.  Suggestion: Lung function monitoring on a respiratory symptoms‐driven basis rather than systematically. |
| “Systemic Juvenile Idiopathic Arthritis-Associated Lung Disease: Characterization and Risk Factors” – Schulert et al., 2019 (35) | Prospective cohort study | 18 patients, “SJIA-LD” – recently recognized albeit poorly defined Lung disease (LD) | - SJIA-LD (ground-glass opacities, subpleural reticulation, interlobular thickening, lymphadenopathy) - patchy but extensive lymphoplasmacytic infiltrates + mixed features of pulmonary alveolar proteinosis (PAP) and endogenous lipoid pneumonia (ELP) - SJIA-LD patients younger than SJIA-patients without LD, prior episodes of macrophage activation syndrome, had adverse reactions to biologic therapy, have higher serum IL-18 | Pulmonary disease is increasingly detected in children with SJIA, particularly in association with MAS. This entity has distinct clinical and immunologic features and represents an uncharacterized inflammatory LD  critical need for well-designed multi-center epidemiologic studies |
| “Spirometric Assessment in Juvenile Idiopathic Arthritis” – Alam et al., 2015 (42) | Cross-sectional study | 33 patients, 6 patients oligoarthritis, 16 polyarthritis, 11 systemic JIA.  17 patients clinically inactive disease, 16 received methotrexate. | - No patient with respiratory symptoms - 13 patients with decreased FVC and normal FEV1/FVC - One with decreased FEV1 and FEV1/FVC with normal FVC - Decreased FEF25-75% in 4 patients - Decreased PEFR in 8 patients | - Abnormal spirometry was present in 13 patients and affected all subsets in terms of subtypes, gender, disease activity and methotrexate therapy - JIA subtypes differed significantly with regard to prevalence of decreased FVC and FEV1 |

**Legend Supplement Table 1.** Clinical studies of pulmonary function in children with juvenile idiopathic arthritis (JIA), emphasizing the effect of disease activity and methotrexate (MTX) treatment. Columns include study details (author, year), study design, patient group, significant pulmonary findings and conclusions regarding disease-related or treatment-related lung involvement.

**Supplement Table 2.**

| **Study** | **Study design** | **Patients** | **Key Findings** | **Conclusion** |
| --- | --- | --- | --- | --- |
| “Hepatotoxicity in patients with juvenile idiopathic arthritis receiving longterm methotrexate therapy” – Lahdenne et al., 2002 (12) | Retrospective cohort study | 34 JIA patients with long-term (>2.4 years) MTX therapy | - Histopathologic findings: All 24 patients on low-dose MTX had grade I histology - Of 10 patients with > 20 mg/m2 MTX, 5 had grade I, 4 had grade II, and 1 had extensive steatosis - No fibrosis or cirrhosis observed | Potential for severe hepatotoxicity of low dose MTX is minimal. Higher MTX doses may increase the risk of histopathologic liver changes. Liver changes, including steatosis, were observed, potentially influenced by corticosteroid use and active inflammation. |
| “Metotrexato en artritis idiopática juvenil: efectos adversos y factores asociados [Methotrexate in juvenile idiopathic arthritis. Adverse effects and associated factors]” – Barral Mena et al., 2020 (13) | Retrospective observational study | 107 JIA patients | - AEs in 48,6% - Gastrointestinal AEs in 35,6% - 20 children required modification of dosage or route of administration, with resolution of AE in 11 cases (55%) - Interruption of MTX therapy in 34,6% | 50 % of patients develop some sort of AE with interruption of therapy in 35 % |
| “SLCO1B1 variants as predictors of methotrexate-related toxicity in children with juvenile idiopathic arthritis” – Roszkiewicz et al., 2021 (39) | Prospective cohort study with additional cross-sectional approach | 100 JIA patients (all subtypes) treated with MTX | - AEs in 28 percent (42,85 % hepatotoxicity) - SLCO1B1 rs4149056 TT variant more likely to develop hepatotoxicity | Genotyping may be used to identify patients at higher risk of AEs linked to MTX treatment and to individualize treatment strategy. |
| “Methotrexate Hepatotoxicity in Children with Juvenile Idiopathic Arthritis: A Single-Center Study” – Yassin et al., 2021 (14) | Observational case control study | 80 JIA patients, 50 with MTX | - Hepatotoxicity definition: at least one value above the normal laboratory range of AST or ALT - 14 patients with hepatotoxicity (28%) - Children with MTX therapy: higher Transaminase interquartile ranges | Hepatotoxicity is a common AE in children with JIA and MTX therapy. |
| “Hepatic and hematological adverse effects of long-term low-dose methotrexate therapy in rheumatoid arthritis: An observational study” – Dubey et al., 2016 (15) | Cross-sectional observational study | 204 RA patients with low-dose MTX therapy (≤15 mg/week) for at least 2 years | - frequency of raised alanine transaminase level (≥3-fold rise above the upper limit of normal) was 6.37% - two biopsy-proven hepatic fibrosis cases | In RA patients, long-term, low-dose MTX therapy is safe in Indian population.  Disease duration, cumulative MTX dose, concomitant DMARD intake are not risk factors associated with hepatic or hematological adverse effects |
| “Methotrexate and hepatic toxicity in rheumatoid arthritis and psoriatic arthritis” – Tilling et al., 2006 (38) | Observational prospective cohort study | 550 RA patients, 69 PsA patients | - both groups with elevated liver enzymes - PsA patients with significantly higher risk of elevated transaminases compared to RA patients (14.5% vs 7.5%) | Methotrexate-treated PsA patients exhibited a higher incidence of hepatotoxicity compared to RA patients treated with methotrexate  Suggestion: Psoriatic patients with higher susceptibility to methotrexate-induced hepatotoxicity than rheumatoid patients. |
| “Methotrexate and psoriasis: 2009 National Psoriasis Foundation Consensus Conference” – Kalb et al., 2009 (11) | Review |  | Consensus on use of MTX in psoriasis (dosing, monitoring, …) | MTX is safe and effective in therapy of psoriasis when selecting and monitoring of patients is carried out properly |

**Legend Supplement Table 2.** Summary of studies investigating hepatotoxicity and adverse hepatic side effects associated with methotrexate (MTX) therapy in juvenile idiopathic arthritis (JIA), rheumatoid arthritis (RA) and psoriatic arthritis (PsA). Columns include study type, patient groups, key hepatic results and conclusions regarding liver safety, risk factors and recommendations during MTX treatment.

**Supplement Table 3.** Comparative Demographic Analysis of Two Investigated Cohorts.

**Legend Supplement Table 3.** Comparative demographic characteristics of the two pediatric cohorts. Summary of patient´s characteristics including JIA subtype distribution, sex ratio, age at disease onset, age at methotrexate (MTX) initiation, treatment duration and the proportion of patients with elevated glutamate pyruvate transaminase (GPT) levels. Entire cohort stratified into two sub cohorts (1993 – 2007 and 2007 – 2023).

Pt: Patient; w/: with; ERA: Enthesitis-related arthritis; RF+/RF-: Rheumatoid factor (RF) positive (+) or negative (-) polyarticular arthritis; Abs#: Absolute Number; GPT: Glutamine-pyruvate transaminase

**Supplement Table 4.** Temporal Analysis of Mean Pulmonary Function in Pediatric Cohort

**Legend Supplement Table 4.** Temporal Analysis of Mean Pulmonary Function in Pediatric Cohort, 1993-2023. This table illustrates the longitudinal mean lung function parameters of children enrolled in the study over the specified period. The time-axis represents the duration of Methotrexate (MTX) treatment in years with the initial pulmonary function test (PFT) conducted at the commencement of MTX therapy denoted as time point 0.

FEV1: Forced expiratory volume; FVC: Forced vital capacity; MMEF: Maximal mid-expiratory flow; TLC: Total lung capacity; RV: Residual volume; DLCO: Diffusion capacity of the lung for carbon monoxide

**Supplement Figure 1**

**
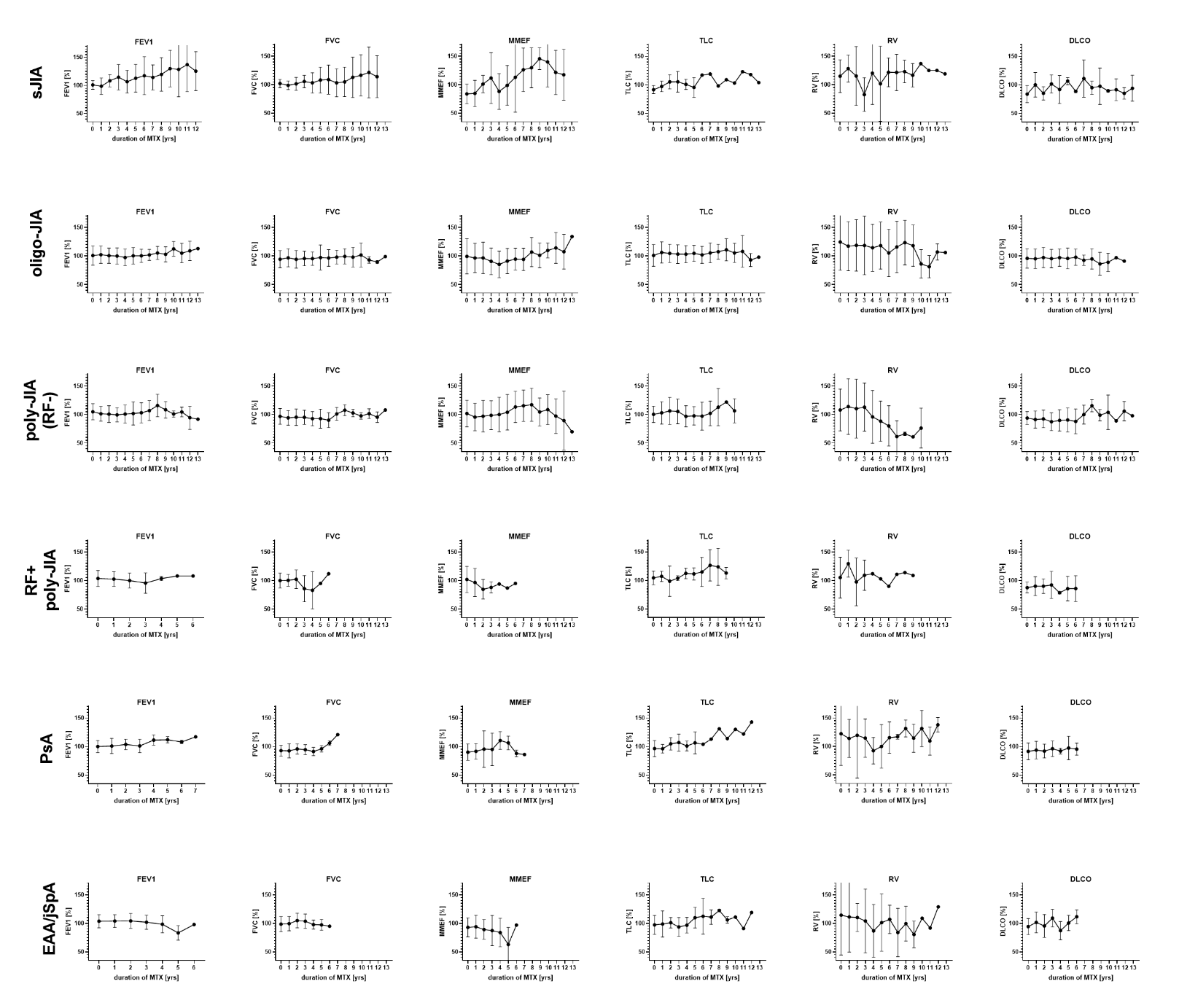
**

**Legend Supplement Figure 1.** JIA-subgroup analysis. Rows: JIA subgroups, columns: Distinct lung function parameter, expressed as percentages. Lines represent mean values (+ standard deviation) of age-specific lung function (y-axis) in the cohort of 274 patients related to treatment duration (x-axis) in years. Paired t-test between comparing each time point with the lung function before starting MTX showed no significant changes over time.

MTX: Methotrexate; sJIA: Systemic JIA (Still's disease); oJIA: Oligoarthritis; polyJIA (RF-): Polyarticular JIA with negative rheumatoid factor; RF+ PolyJIA: Polyarticular JIA with positive rheumatoid factor; PsA: Psoriatic JIA; EAA/SpA: Enthesitis-associated arthritis/spondyloarthropathy.FEV1: Forced expiratory volume in 1 second; FVC: Forced vital capacity; MMEF: Maximum mid-expiratory flow; TLC: Total lung capacity; RV: Residual volume; DLCO: Diffusing capacity for carbon monoxide.

**Supplement Figure 2:**

**Legend Supplement Figure 2.**

**Duration of Methotrexate (MTX) Treatment Until Discontinuation by Cause**
Kaplan–Meier curves illustrating the time to MTX discontinuation stratified by documented reason. Total counts per category were: remission (n = 82), elevated liver enzymes (ELE; n = 17), gastrointestinal intolerance (GI; predominantly nausea; n = 10), non-compliance (n = 9) and abnormalities in complete blood count (CBC; n = 2). The remission curve (black) shows the longest treatment durations, whereas toxicity-related discontinuations (ELE, GI, CBC) occurred earlier, typically within the first 24–36 months. ELE = elevated liver enzymes; CBC = hematologic abnormalities; GI = gastrointestinal intolerance.
